# Supplementary material for: Integrated analysis of the aqueous humor microbiome and lens capsule transcriptome in high myopia cataract: a pilot study
Source: Front Med (Lausanne). 2026 Jun 16;13:1845205. doi: 10.3389/fmed.2026.1845205 (PMC13314463; doi:10.3389/fmed.2026.1845205)
Supplement: Supplementary file 3 [file Data_Sheet_1.zip › 4.Meta_Statistic/C372089/T_test/Genus/diff_Genus_top10_boxplot_each2.pdf]

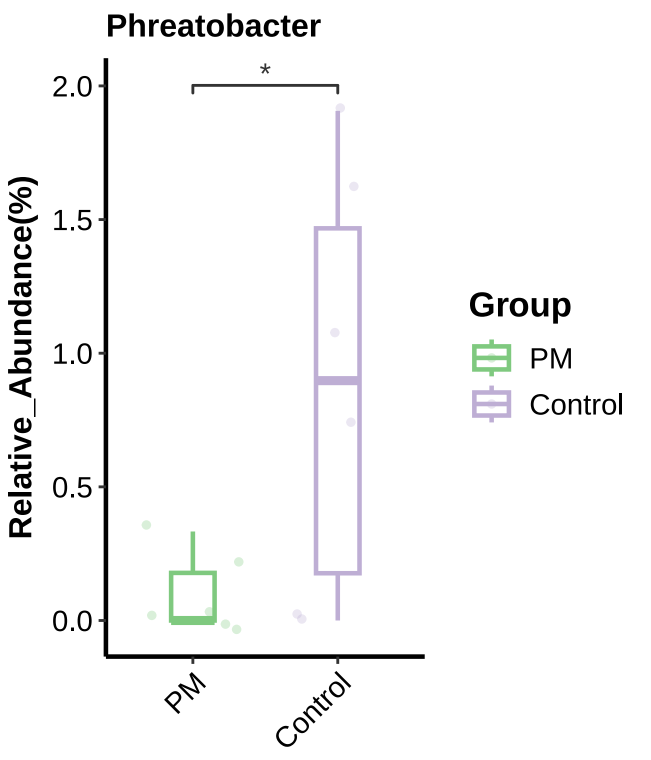
Phreatobacter
*
2.0
1.5
1.0
0.5
0.0
Relative_Abundance(%)
PM
Control
Group
PM
Control
